# Soccer Practice and Functional and Social Performance of Men With Lower Limb Amputations

**DOI:** 10.2478/hukin-2014-0087

**Published:** 2014-11-12

**Authors:** Rogeria Monteiro, Luzia Pfeifer, Alex Santos, Nelson Sousa

**Affiliations:** 1 Department of Occupational Therapy, Pará State University, Belém, Brazil.; 2 Department of Neuroscience and Behavioral Sciences, Division of Occupational Therapy, School of Medicine, São Paulo University at Ribeirão Preto, Ribeirão Preto, Brazil.; 3 Biostatistics Institute, Belém, Brazil.; 4 Research Center in Sport Sciences, Health Sciences and Human Development, University of Trás-os-Montes e Alto Douro, Vila Real, Portugal.

**Keywords:** amputee soccer, occupational performance, international classification of functionality, disability and health

## Abstract

Practicing sports together with rehabilitative treatment improves the development of motor, social and emotional abilities of lower limb amputees. The aim of this study was to compare the functional and social performance of individuals with lower limb amputations between those who played soccer and those who did not engage in any sports activities. A total of 138 individuals participated in the study and were divided into two groups: soccer players (n = 69, 34 ± 8.1 years) and non-athletes (n = 69, 38 ± 8.9 years). A checklist, based on the International Classification of Functioning, Disability and Health, was used. Data were analyzed using the Chi-square and Mann-Whitney tests. The soccer players group showed significantly better performance than the non-athletes group in most items of body function, body structure, occupational performance components and daily activities (p < 0.001 for all), and also in some important items of social and environment factors (p < 0.001 for all). The results strongly suggest that amputee soccer significantly improves the functional and social performance in individuals with lower limb amputations.

## Introduction

Individuals with amputations may experience related complications and traumas, such as phantom sensations and pain, amputation site pain, painful neuromas, dermatological changes, ulcerations and infections (Probstner and Thuler, 2006). Other effects include decreased muscular strength and hypotrophy, changes in muscle tone, edema, changes in posture, changes in sensitivity, reduction in the range of motion, changes in motor coordination and balance (Keenan and Morris, 2004). The sudden disruption of mobility or loss of body parts may result in changes related to body image. Such disorders may lead to the loss of or dysfunctional self-esteem, and may interfere with the performance of daily life activities, such as work or social roles (Yuen and Hanson, 2002; Pedretti and Early, 2004).

Rehabilitative treatment should be initiated early on and involve an interdisciplinary team that includes an orthopedist, vascular surgeon, physiatrist, physical therapist, occupational therapist, psychologist, nurse, nutritionist among others (Chamlian, 2004). The main objective of a rehabilitative process is to enable the amputee to recover functional independence, involving cognitive, emotional and behavioral adaptations (Alavaro and Wald, 2004). Adapted sports contribute to the improvement of daily life activities, and may contribute to the development of a more positive perception of the body in the amputees (Sousa et al., 2009). Some studies have shown that practicing adapted sports together with rehabilitative treatment improves the development of motor, social and emotional abilities (Garvey, 1989).

Currently, various adapted sports are available to people with disabilities, and the literature has shown the benefits of their practice. Amputee soccer stands out among such sports (Gomes et al., 2005). To the best of our knowledge, there are only few studies addressing the potential benefits of amputee soccer, and among those who have addressed it, none analyzed the influence on the functional performance of amputees (Gomes et al., 2005; Lowther et al., 2002; Simim et al., 2010). Therefore, the aim of this study was to compare the functional and social performance of individuals with lower limb amputations between those who played amputee soccer and those who did not engage in any sport activities.

## Material and Methods

### Experimental Approach to the Problem

This cross-sectional, non-experimental quantitative study was conducted to compare two groups: the group of soccer practitioners and the group of non-practitioners of any adapted sport.

The functional and social performance checklist for lower limb individuals with amputation (DSF) (Monteiro et al., 2013), composed by 108 items based on the International Classification of Functionality, Disability and Health (ICF), was used. This instrument includes body functions and structures (Organic Aspects), activity and participation (Daily Tasks, Performance Components, Social Participation) and Environmental Factors.

### Subjects

The study population comprised 138 adult males with unilateral lower limb amputation. The participants were divided into two groups: the group of soccer practitioners (GSP, n = 69) and the non-practitioners control group (NCG, n = 69).

The causes of amputation were reported by the participants: 77% of the causes reported by the GSP and 67% reported by the NCG were trauma-related. The GSP did not report any co-morbidity, while three individuals in the NCG reported experiencing co-morbidities. In terms of the educational level, most of the individuals in the GSP had graduated secondary school (53.6%), followed by those who completed primary school (14.5%). A small number of individuals in the GSP was enrolled in college (7.2%) or had already attained a bachelor’s degree (8.7%). Many of the individuals in the NCG had not completed primary school (31.9% vs. 24.6% of individuals who had completed primary school), followed by those who had graduated secondary school (29.0%). Six individuals in the NCG and three in the GSP were already retired, while the remaining (63 in the NCG and 66 in the GSP) had a professional activity. In regard to the individuals’ occupations, those in the GSP predominantly worked with sales and services followed by activities in the administrative area. Sales and services also predominated in the NCG, followed by activities in maintenance and repair. It is worth noting that one individual in the GSP and nine in the NCG were on sick leave receiving welfare payments.

Informed consent was obtained prior the study from all subjects. Ethics approval was received from the Ethics Research Committee at Hospital in the same city where the research was carried out (Protocol n^º^. 065/09).

### Procedures

Data were collected in 12 cities from seven Brazilian states, along with the Federal District. Interviews took 50 min, on average. The DSF was applied, individually with each participant, by a researcher who read the checklist. The checklist had the script that included some direct questions concerning body functions as well as body structures and environmental factors.

Data were collected in a private environment which reduced physical and auditory distractions to facilitate the completion of the questionnaires by researchers.

One card containing the ICF qualifiers was handed to each participant to make it easy to answer the questions. The activities and participations could be qualified at 6 levels being 0 (if the participant had no difficulty), 1 (if the participant had mild difficulty), 2 (if the participant had moderate difficulty), 3 (if the participant had severe difficulty), 4 (if the participant was not able to proceed taking into account the level of difficulty), 8 (if there was insufficient information to specify the level of difficulty) and 9 (if not applicable to this case). The environmental factors could be qualified at 6 levels being 0 (if the participant recognized no barriers) and + 0 (if the participant recognized that there was no facilitator); 1 (if the participant recognized to have a mild barrier), + 1 (if the participant recognized to have a mild facilitator); 2 (if the participant recognized to have a moderate barrier), + 2 (if the participant recognized to have a moderate facilitator); 3 (if the participant recognized to have a severe barrier), + 3 (if the participant recognized to have an extensive facilitator); 4 (if the participant was not able to proceed taking into account the barrier), and + 4 (if the participant was not able to proceed taking into account the facilitator); 8 (if there was insufficient information to specify the severity of the barrier), + 8 (if there was insufficient information to specify the severity of the facilitator); 9 (if not applicable to this case).

### Statistical Analysis

The items of the DSF checklist were grouped into five domains: Domain 1 – Organic aspects; Domain 2 – Daily activities; Domain 3 – Performance components; Domain 4 – Social participation; and Domain 5 – Environmental factors (Monteiro et al., 2013).

Statistical analysis checked for potential differences between the two groups in terms of functional and social performance. The distribution of characteristics was observed between the groups and the Pearson’s Chi-square test was applied. The Mann-Whitney test was used to compare scores between the two independent groups (Siegel and Castellan, 2006).

Descriptive statistics were used to present qualitative variables through proportional distributions, while quantitative variables were presented through a central tendency and variation measures. The Chi-square test was applied as a method of statistical inference used to compare performance between the two groups. Due to a restriction (npq<5), the G test was used as recommended by Ayres et al. (2007). A comparison between quantitative variables was performed by the Mann Whitney test, since the D’Agostino-Person test indicated that samples did not present a Gaussian distribution (Siegel and Castellan, 2006). The alpha level was fixed at p<0.01 to reject the null hypothesis, aiming to identify which items in the questionnaire presented the most extreme differences between the two groups being compared. BioEstat version 5.2 was used in the entire statistical process.

## Results

### The general characteristics of the GSP and NCG

The average age of the individuals composing the GSP was 34 ± 8.1 years, while the average age among those in the NCG was 38 ± 8.9 years; no statistical difference was observed between groups. A significant difference (p = 0.0015) was observed in relation to time since amputation. The average time since amputation among the individuals in the GSP was higher (15.4 ± 10.5 years) than in the NCG, whose individuals had an amputation for an average of 10.5 ± 9.9 years. The point where the limb was amputated was higher for the GSP: transfemoral amputations were identified in 76.8% of the cases while thigh amputations were observed in 65.2% of the NCG subjects (p < 0.001).

### Comparisons of the functional and social performance according to the domain

Domain 1 – Body features. This domain addressed ICF items concerning body function and body structures. A significant difference (p < 0.001) was observed between groups in this domain, i.e. the number of individuals who reported any deficiency in the GSP was considerably higher when compared with the number of individuals in the NCG.

Domain 2 – Daily activities. The second domain was composed of 35 ICF items and referred to activity and participation (general tasks and demands, making purchases, such as those necessary to live, domestic life, major life areas, self-care and mobility). Significant differences were observed between groups concerning 29 items in this domain (p < 0.001 for 29 items). The number of individuals in the GSP reporting no deficiency was considerably higher than that in the NCG. Distribution of categories concerning the daily activities domain is presented in Table 1.

Domain 3 – Performance components. This domain was composed of items that also involved activity and participation, specifically mobility (transporting, moving and handling objects, changing and maintaining the body’s position, moving objects with the lower limbs, being able to reposition). Significant differences were found between groups in 17 out of 18 items in this domain (p < 0.001 for 17 items). The number of individuals in the GSP reporting no difficulties was considerably higher than that in the NCG. Distribution of categories concerning the performance components domain is presented in Table 2.

Domain 4 – Social participation. The fourth domain comprised the last ICF areas of activity and participation (interpersonal interactions and relationships, major life areas, community, social and civic life). Significant differences were found in 14 out of the 31 items, i.e. the number of individuals in the GSP reporting no difficulties in this domain was significantly higher than in the NCG. Significant differences were found in romantic relationships (p < 0.001), sexual relationships (p = 0.001), informal education (p < 0.001), school education (p < 0.0001), higher education (p < 0.001), seeking employment (p < 0.001), part-time jobs (p < 0.0011), full-time jobs (p < 0.001), formal associations (p < 0.001), ceremonies (p < 0.001), playing (p < 0.001), practicing sports (p < 0.001), art and culture (p = 0.001) and organized religion (p = 0.006).

**Figure 1:**
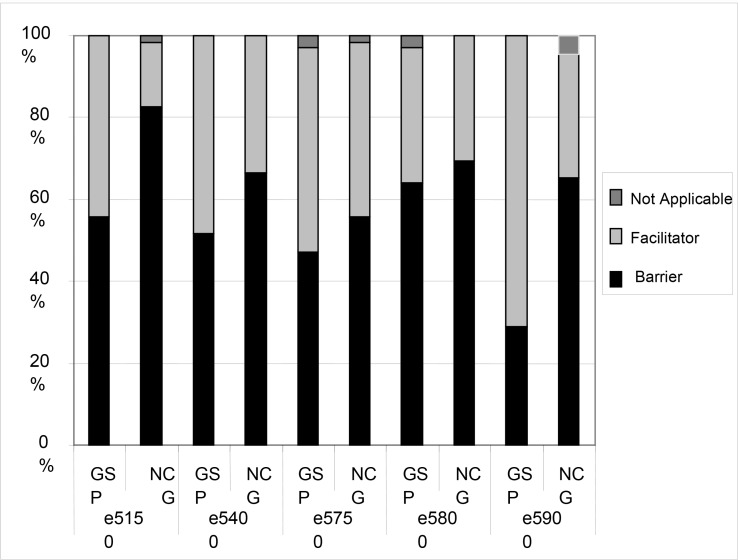
Relative frequencies (%) of the environmental factors’ domain categories (services, systems and policies) in the group of soccer practitioners (GSP, n = 69) and the non-practitioners control group (NCG, n = 69).

Domain 5 – Environmental factors. This last domain was composed of 18 items concerning the ICF environmental factors. Significant differences were observed in 4 items, i.e. the number of individuals reporting no barrier was considerably higher in the GSP compared to the NCG in the following: products and technologies for cultural, recreational and sport activities (p < 0.001); support from acquaintances, colleagues, neighbors and members from the community (p < 0.001); services related to architecture and construction (p < 0.001); and services related to work and employment (p < 0.001). No differences were found in support products and technologies for personal use in daily life, general products and technologies for mobility and personal transportation in indoor and outdoor environments, general products and technologies to work, products and technologies of assistance to work, architecture, construction and materials and architectonic technologies in buildings for public use, architecture, construction, materials and architectonic technologies in the accesses of inner facilities of buildings for public use, support for near family, support for health professionals, individual attitudes of close family members, individual attitudes of acquaintances, partners, colleagues, neighbors and members of the community, services related to transportation, services related to social support in general, services related to health. In addition, environmental factors were considered a facilitator by more subjects in the GSP than in the NCG for most of the items in this domain (Figure 1).

Compared to the NCG, the individuals in the GSP showed a significantly better performance in 70 (64%) out of the 108 instrument’s items. It is remarkable that among the 38 items in which differences between GSP and NCG subjects were not significant, 6 (5.5%) were related to Domain 2 – daily activities, 1 (1.0%) item was related to Domain 3 – performance items, 17 (17.5%) items were related to Domain 4 – social participation, and 14 (14.5%) items were related to Domain 5 – environmental factors.

## Discussion

Although the individuals in the GSP had experienced a longer average time since amputation and also experienced a higher level of amputation compared to the NCG, the number of participants in this group who reported having no difficulties in performing daily activities was significantly higher than in the NCG. The differences between the groups related to Domain 1, which dealt with body function and body structure, may suggest that amputee soccer contributed to a better functional performance.

Both body functions (range of motion and muscular strength) and motor requirements for occupational performance (maintaining different postures, making changes in posture, moving in indoor and outdoor environments, bypassing obstacles, having the ability to transport objects, going up and down stairs, jumping, running, etc.) are developed while playing amputee soccer (Yazicioglu et al., 2007). These abilities can be acquired during training, in the course of tournaments or during other activities inherent to an athlete who needs to travel by air or land, travel different distances to reach training locations, using bilateral crutches while playing (Gomes et al., 2005) and reconciling the athlete’s life with other productive activities.

Thus, all requirements are reflected not only in the physical conditioning of amputees, but also in how their performance of other daily activities improves (Marques et al., 2009; Rosadas, 1986). The subjects of the GSP presented significantly higher results concerning functional independence compared to the NCG, and similar to the results found in the study of Yazicioglu et al. (2007).

As for Social Participation (Domain 4), only 14 of the 31 components showed significant differences in response between the two groups, where the GSP reported better social performance in romantic and sexual relationships, educational and work activities, and participation in physical activities. Relationships in general, are indirectly associated with self-esteem and body image (Maia, 2010; Galhordas and Lima, 2004). In addition, the results of the present study suggest that amputee soccer, as a group activity, enhances independence, initiative, ability to handle new situations and frustrations, and also the will in overcoming obstacles, reflecting a greater ability in daily experiences of the individuals (Marques et al., 2009; Melo and Lopez, 2002; Souza, 1994; Sade and Chacon, 2008).

In Domain 5 (Environmental Factors), again, the GSP showed a significant facilitator perception in some important environment factors, such as recreational and sport activities, community member and architecture, compared to the NCG. Thus, the present results suggest that practicing amputee soccer may contribute to reduce the environment barriers. Apparently, athletes with lower limb amputation appeared to be more proactive in searching for a solution, and appeared less discouraged by failing, as demonstrated in a recent study by Bragaru et al. (2013).

Even though, it is noteworthy that no significant differences were observed between the GSP and NCG in some important items of the psychosocial benefits accruing from sports practice. Such benefits have been reported in other studies and included social inclusion, increased self-esteem, personal growth, the ability to face and overcome challenges, and a reduced level of aggression (Marques et al., 2009; Rosadas, 1986; Melo and Lòpez, 2002).

The study’s limitations are related to the fact that the individuals’ life histories were not taken into account, whether before amputation or before they started practicing amputee soccer. The second limitation is related to the fact that individuals were interviewed only once. Obviously, a longitudinal study would enable monitoring the individuals over a period of time and identifying potential changes. Nonetheless, many limitations are imposed on a study addressing a relatively large population distributed across various states in a large country such as Brazil.

## Conclusion

The results strongly suggest that amputee soccer significantly improves the functional and social performance in individuals with lower limb amputations. Nonetheless, a cross-sectional study does not provide enough support to state that the practice of soccer is the only cause of such improvements. Therefore, further studies are needed to investigate this hypothesis.

## Practical Applications

This study allowed us to compare the functional and social performance of individuals with lower limb amputation that are practitioners and non-practitioners of amputee soccer. The results of the present study support the idea that participation in adapted sports, by individuals with lower limbs amputation, is a privileged means to promote functional and social rehabilitation.

## Figures and Tables

**Table 1:** Distribution of categories concerning the daily activities domain (self-care, walking and moving) between the group of soccer practitioners (GSP, n = 69) and the non-practitioners control group (NCG, n = 69).

		**None**	**Slight**	**Moderate**	**Extensive**	**Complete**	**Not Specified**	**Not Applicable**	**p-value**
d5101- Washing the entire body	GSP	94%	6%	0%	0%	0%	0%	0%	< 0.001
	NCG	67%	13%	19%	1%	0%	0%	0%	
d5200 – Skin care	GSP	94%	6%	0%	0%	0%	0%	0%	< 0.001
	NCG	67%	13%	19%	1%	0%	0%	0%	
d5202- Hair and facial hair care	GSP	97%	1%	0%	0%	0%	0%	1%	0.051
	NCG	84%	6%	6%	3%	1%	0%	0%	
d5204-Toenail care	GSP	96%	3%	1%	0%	0%	0%	0%	< 0.001
	NCG	61%	7%	10%	12%	3%	1%	6%	
d5400 –Dressing	GSP	84%	14%	1%	0%	0%	0%	0%	< 0.001
	NCG	49%	32%	14%	3%	1%	0%	0%	
d5401-Undressing	GSP	84%	14%	1%	0%	0%	0%	0%	< 0.001
	NCG	49%	28%	14%	6%	3%	0%	0%	
d5402 –Putting on shoes	GSP	97%	1%	1%	0%	0%	0%	0%	< 0.001
	NCG	57%	16%	9%	16%	3%	0%	0%	
d5403-Taking off shoes	GSP	99%	1%	0%	0%	0%	0%	0%	< 0.001
	NCG	59%	16%	7%	14%	3%	0%	0%	
d5702-Maintaining health	GSP	93%	4%	1%	0%	0%	0%	1%	< 0.001
	NCG	51%	10%	23%	12%	3%	0%	1%	
d4500 –Walking short distances	GSP	96%	1%	0%	1%	1%	0%	0%	< 0.001
	NCG	61%	17%	14%	4%	3%	0%	0%	
d4501-Walking long distances	GSP	43%	25%	22%	10%	0%	0%	0%	< 0.001
	NCG	17%	6%	23%	19%	33%	1%	0%	
d4502-Walking over different surfaces	GSP	38%	35%	19%	7%	1%	0%	0%	< 0.001
	NCG	9%	17%	29%	28%	16%	0%	1%	
d4503-Walking around obstacles	GSP	72%	14%	12%	1%	0%	0%	0%	< 0.001
	NCG	38%	13%	12%	16%	19%	0%	3%	
d4600 –Moving about within the house	GSP	99%	0%	1%	0%	0%	0%	0%	< 0.001
	NCG	64%	13%	13%	6%	4%	0%	0%	
d4601 - Moving within buildings other than one’s own house	GSP	81%	13%	6%	0%	0%	0%	0%	< 0.001
	NCG	45%	22%	25%	6%	3%	0%	0%	
d4602 - Moving outside of the house and other buildings	GSP	78%	14%	4%	1%	0%	1%	0%	< 0.001
	NCG	51%	12%	22%	10%	3%	0%	3%	
d465 - Moving using some type of equipment	GSP	84%	7%	4%	1%	3%	0%	0%	< 0.001
	NCG	42%	17%	17%	12%	3%	0%	0%	

* Between groups.

**Table 2:** Distribution of the performance components domain (changing and maintaining the body systems) among the group of soccer practitioners (GSP, n = 69) and the non-practitioners control group (NCG, n = 69).

		**None**	**Slight**	**Moderate**	**Extensive**	**Complete**	**Not Specified**	**Not Applicable**	p-value*
d4100-Lying down	GSP	97%*	3%	0%	0%	0%	0%	0%	< 0.001
	NC	58%	25%*	14%*	3%	0%	0%	0%	
d4101-Crouching	GSP	67%	19%	7%	4%	3%	0%	0%	< 0.001
	NCG	16%	12%	26%	19%	26%	0%	1%	
d4102-Kneeling	GSP	59%	13%	16%	4%	1%	0%	6%	< 0.001
	NCG	22%	10%	19%	23%	19%	3%	4%	
d4103-Sitting down	GSP	86%	14%	0%	0%	0%	0%	0%	< 0.001
	NCG	46%	29%	16%	6%	3%	0%	0%	
d4104-Standing up	GSP	81%	17%	1%	0%	0%	0%	0%	< 0.001
	NCG	32%	33%	23%	9%	3%	0%	0%	
d4105-Leaning	GSP	86%	13%	0%	1%	0%	0%	0%	< 0.001
	NCG	28%	23%	22%	12%	14%	1%	0%	
d4106-Changing the body’s centre of gravity	GSP	70%	17%	7%	1%	3%	0%	1%	< 0.001
	NCG	30%	16%	17%	13%	22%	1%	0%	
d4154 –Remaining standing	GSP	67%	25%	6%	3%	0%	0%	0%	< 0.001
	NCG	28%	19%	28%	17%	9%	0%	0%	
d4200-Moving while seated	GSP	91%	6%	3%	0%	0%	0%	0%	< 0.001
	NCG	64%	13%	10%	9%	4%	0%	0%	
d4300-Lifting objects	GSP	71%	13%	16%	0%	0%	0%	0%	< 0.001
	NCG	49%	16%	14%	9%	7%	1%	3%	
d4301-Moving objects in one’s hands	GSP	46%	36%	16%	1%	0%	0%	0%	< 0.001
	NCG	30%	12%	17%	20%	19%	0%	1%	
d4302-Moving objects in one’s arms	GSP	49%	29%	9%	9%	1%	1%	1%	< 0.001
	NCG	35%	12%	10%	23%	17%	0%	3%	
d4305-Placing objects	GSP	81%	12%	7%	0%	0%	0%	0%	0.185
	NCG	70%	13%	13%	4%	0%	0%	0%	
d4350-Pushing with the lower limbs	GSP	55%	12%	10%	6%	6%	0%	12%	0.001
	NCG	17%	10%	9%	12%	19%	13%	20%	
d4351-Kicking	GSP	90%	3%	4%	0%	3%	0%	0%	< 0.001
	NCG	42%	6%	14%	12%	17%	4%	4%	
d4551-Going up/down stairs	GSP	65%	16%	14%	3%	1%	0%	0%	< 0.001
	NCG	23%	10%	23%	30%	10%	0%	3%	
d4552-Running	GSP	74%	14%	9%	0%	3%	0%	0%	< 0.001
	NCG	4%	6%	4%	13%	65%	4%	3%	
d4553-Jumping	GSP	74%	9%	17%	0%	0%	0%	0%	< 0.001
	NCG	20%	12%	14%	12%	38%	1%	3%	

* Between groups.
